# Psychotherapy training in Turkey and Europe, similarities and differences

**DOI:** 10.1192/j.eurpsy.2025.167

**Published:** 2025-08-26

**Authors:** E. Sönmez Güngör

**Affiliations:** Psychiatry, Erenköy Mental Health and Neurological Diseases Training and Research Hospital, Istanbul, Türkiye

## Abstract

**Abstract:**

Evidence for efficacy of various modalities of psychotherapies is growing and such therapies are increasingly recommended in international guidelines for the treatment of psychiatric disorders. Psychiatrists with strong psychotherapy skills are better positioned to provide individualized and multidisciplinary care. Training in psychotherapy also fosters the development of reflective practice, empathy, and cultural competence, all of which are vital in addressing the diverse needs of patients. Accessibility of psychotherapeutic treatment for the patients can be improved through integrating psychotherapy training in psychiatric training, together with continuous medial education activities. European and other international organisations have published guidelines requiring programs to promote psychotherapeutic competences among psychiatry trainees. However, psychotherapy education and supervision can often become a luxury rather than being a mandatory component of training; and resources are heterogenous. In this presentation, psychotherapy training availabilities and limitations in Turkey will be discussed, with a focus on the psychotherapy courses of Psychiatric Association of Turkey among other initiatives.

**Disclosure of Interest:**

None Declared

